# Patient specific deep learning based segmentation for magnetic resonance guided prostate radiotherapy

**DOI:** 10.1016/j.phro.2022.06.001

**Published:** 2022-06-03

**Authors:** Samuel Fransson, David Tilly, Robin Strand

**Affiliations:** a Department of Medical Physics, Uppsala University Hospital, Uppsala, Sweden; b Department of Surgical Sciences, Uppsala University, Uppsala, Sweden; c Department of Immunology, Genetics and Pathology, Uppsala University, Uppsala, Sweden; d Department of Information Technology, Uppsala University, Uppsala, Sweden

## Abstract

**Background and Purpose:**

Treatments on combined Magnetic Resonance (MR) scanners and Linear Accelerators (Linacs) for radiotherapy, called MR-Linacs, often require daily contouring. Currently, deformable image registration (DIR) algorithms propagate contours from reference scans, however large shape and size changes can be troublesome. Artificial neural network (ANN) based contouring may alleviate this issue, however generally requires large datasets for training. Mitigating the problem of scarcity of data, we propose patient specific networks trained on a single dataset for each patient, for contouring onto the following datasets in an adaptive MR-Linacworkflow.

**Materials and Methods:**

MR-scans from 17 prostate patients treated on an MR-Linac with contours of Clinical Target Volume (CTV), bladder and rectum were utilized. U-net shaped models were trained based on the image from the first fraction of each patient, and subsequently applied onto the following treatment images. Results were compared with manual contours in terms of the Dice coefficient and Added Path Length (APL). As benchmark, contours propagated through the clinical DIR algorithm were similarly evaluated.

**Results:**

In Dice coefficient the ANN output was 0.92 ± 0.03, 0.93 ± 0.07 and 0.84 ± 0.10 while for DIR 0.95 ± 0.03, 0.93 ± 0.08, 0.88 ± 0.06 for CTV, bladder and rectum respectively. Similarly, APL where 3109 ± 1642, 7250 ± 4234 and 5041 ± 2666 for ANN and 1835 ± 1621, 7236 ± 4287 and 4170 ± 2920 voxels for DIR.

**Conclusions:**

Patient specific ANN models trained on images from the first fraction of a prostate MR-Linac treatment showed similar accuracy when applied to the subsequent fraction images as a clinically implemented DIR method.

## 1 Introduction

Magnetic Resonance (MR) imaging provides high soft-tissue contrast without using ionizing radiation, and since the introduction of devices combining MR-scanners and Linear Accelerators, called MR-Linacs, the radiotherapy field has moved to adaptive workflows. Daily high-quality soft-tissue imaging with the integrated MR, and replanning while the patient is in treatment position, enables dose delivery tailored to the daily anatomy. Daily replanning on an MR-Linac [1] system includes the full radiotherapy workflow, compressed into generally less than an hour, with image fusion to a reference image, recontouring of the anatomy and replanning based on the new contoured anatomy, and execution of the delivery. The workflow is very resource intensive and one of the most time consuming aspects of the replanning workflow is the recontouring step ([2] as well as identified in our clinic) where both the target and relevant organs at risk (OARs) need to be recontoured.

Deformable image registration (DIR) has become a mainstream solution for contour propagation [3] with mixed results depending on both anatomical site and magnitude of anatomy changes [[4], [5], [6]]. Interfractional variations in both bladder and rectum in prostate radiotherapy patients may be substantial and the algorithm may struggle to produce accurate and smooth contours, requiring the physician to manually adjust (or completely recontour) the organ under time pressure with the patient in treatment position. More complex solutions to handle large interfractional changes have been suggested, e.g. introducing deep learning based contours into the DIR pipeline [7], and with methods directed towards the MR-Linac treatment workflow such as using multiple DIRs to propagate contours from previous treatment sessions and combining into consensus contours [8]. A deep learning network jointly obtaining contour propagation and a deformation field for CTV (Clinical Target Volume) in an MR-Linac setting has been proposed [9]. Contour propagation through DIR for intrafractional motion has also been investigated for prostate MR-Linac treatments [10].

Artificial neural networks (ANNs) for semantic segmentation are now present for a variety of medical image modalities and anatomical regions [11]. Training deep learning networks generally requires a large image cohort with consistent contours on same image modality, hence for medical purposes training to contour OARs on CT-images is generally the most straightforward approach. These ANNs should learn general features of the training dataset for reliable behavior during inference, i.e. to be able to accurately produce contours onto a never seen dataset of a new patient, since in conventional radiotherapy there is only a necessity to contour on a single dataset for each patient. However, the longitudinal character of an MR-Linac treatment infers the necessity to contour the images each fraction and hence the possibility to utilize daily images for training of a patient specific ANN. ANNs also almost exclusively contour the OARs, excluding the definition of the target, since the target is not necessarily limited to a specific organ but rather pathologically determined and therefore can vary between patients.

Patient specific networks would therefore be a suitable choice since the models will be agnostic to patient specific target definitions as well as potential abnormal patient specific anatomy not present in the training data (e.g extensive surgical resections) as well as to MR imaging protocol. This can also circumvent the otherwise dominant issue of requiring large dataset for general models. While not making much sense for a conventional external therapy workflow due to above reasoning of one-time contouring, it is suitable for an adaptive workflow requiring adaptations on a daily basis. Utilizing transfer learning through networks trained on a large image dataset such as Imagenet [12] is a potential approach, however choosing how and which layers to update is a challenging and highly critical task [13], not guaranteed to improve results compared to training from scratch [14]. A transfer learning approach for fine-tuning on a patient-level on prostate CT-scans in an adaptive approach has been performed [15], and similarly on MR-images [16]. However, such approaches require a pretrained model to begin with.

In the context of an MR-Linac workflow, our aim was to examine the possibility to train patient specific ANNs from scratch with only a single 3D-image and evaluate its accuracy on the upcoming fractions, including both target and relevant OARs. We benchmarked it against the already implemented DIR method in the treatment planning system to evaluate the potential applicability.

## 2 Material and methods

The MR-Linac workflow used (shown schematically in Fig S1 in Supplementary Material) consisted of acquiring a reference MR-image before the first fraction (offline) from a conventional 3T MR on which the target along with all relevant OARs were contoured. A treatment plan was made and approved acting as a reference plan for adaptation at the upcoming treatments (online). The contours were then propagated online through a DIR algorithm onto the daily image set and manually adjusted, or completely recontoured by a physician, depending on the accuracy of the contour propagation. A verification scan was acquired to identify any potential motion during the previous steps during the plan adaptation. The plan was then approved and delivered during which an additional scan was acquired.

### 2.1 Dataset

Images from ultrahypofractionated treatments of prostate patients, 6.1 Gy × 7 fractions were available for the study. All data used herein was preceded by informed consent and with approval from the Swedish Ethical Review Authority (2019–03050). The planning images were acquired through the on scanner pre-defined exams, yielding T2-weighted transversal images with 0.86 × 0.86 × 1 mm resolution with 2 min acquisition time. At treatment the CTV was accurately contoured as well as the OARs in the vicinity of the CTV. Retrospectively however images were more fully contoured including the CTV, bladder and rectum. Seventeen patients with associated scans were available. However, some fractions were delivered on either conventional accelerators or with a workflow not requiring recontouring on the MR-Linac and resulted in a total of 112 fraction images available for the study. The CTV varied between patients, encompassing the whole prostate and sometimes also the seminal vesicles.

### 2.2 Network design and training

The images from the first fraction for each patient was set as the training data for the ANN framework. The images were cropped to contain the contoured structures with a minimum size of 256 × 256 × 128 voxels in the left–right, anterior-posterior and feet-head direction, to speed up training and reduce both the memory requirements as well as class imbalance due to the otherwise large amount of background voxels. A 2D U-net-shaped model was employed on the data along the transversal axis and the model was saved every 100th epoch during a total training of 1000 epochs (see Supplementary Material for more detailed description of architecture). Of the 17 patients, 3 were used for internal validation of the network design finding e.g. appropriate depth and learning rate, leaving 14 patients for actual testing of the workflow. Batch size was set to 4 (the small size attributed to the highly limited number of training images), SeLu (Scaled exponential Linear unit) activation [17] employed in the hidden layers and 4 probability maps (background, CTV, bladder and rectum) obtained through softmax activation of the last layer. Training was performed with random 128 × 128 patches extracted from the images to augment the small dataset. The Adam optimizer was used for training with a learning rate of 1e-4. The training was implemented in Tensorflow 2.4 and performed on an Nvidia RTX 3070. The reference dataset with corresponding structures (see Fig S1 in Supplementary Material for explanation) was used as validation data during training to observe proper convergence and also notice potential overfitting. To further improve results and to prevent overfitting small random deformations along with noise and contrast augmentations were employed as well as alpha dropout of 0.4 in the bottom layer. The network was trained to simultaneously segment the CTV, bladder and rectum. Despite cropping a quite noticeable class imbalance was identified (∼93% background voxels) hence a focal loss implementation [18] with gamma = 2 was utilized.

At inference a horizontal ensemble approach [19] was utilized with the three last saved models where the results from each of the networks underwent a soft voting procedure producing the final segmentation map. This was performed on all of the remaining fractional images for each patient, i.e. fraction 2 and forward. The inference of the ensemble approach was thought to stabilize end results [19] and remove any spurious noise and small misclassifications, which could be expected due to the limited amount of training data. Postprocessing of the results included keeping only the largest coherent structure for each output, removing any potential still remaining noise.

### 2.3 Evaluation measures

As evaluation measures the commonly applied Dice coefficient overlap was utilized, which is a pure geometric measure of segmentation overlaps. However, its correlation to the recontouring time is not optimal [20], which in the sense of online contour propagation is arguably more important. The Added Path Length (APL) that has been shown to have a higher correlation to recontouring time [21] was therefore added as evaluation measure. It is a measure of how much of the total length of the generated contours that are beyond a tolerance from the reference contours, hence indicating how much requires recontouring. Larger values of APL therefore indicate longer recontouring time. A one voxel tolerance was chosen, not considering the voxel anisotropy. The output of the ANNs were compared to the reference contours for a direct measurement of the ANN segmentation accuracy. Also, it was benchmarked against the results from the current DIR algorithm in the treatment planning system (with the same reference image as in the ANN training). A Wilcoxon two-sided rank test was employed to depict significance levels between the ANN and DIR approach. This test was chosen since we did not assume normal distribution.

## 3 Results

In total, the DIR approach produced slightly better results than the ANN in terms of Dice coefficient and APL for the CTV and rectum with a statistically significant difference (p < 0.05) while performance for the bladder was similar, however with a difference not statistically significant (p > 0.05), see Table 1.

**Table 1:** Mean values and standard deviations of the output of ANN (Artificial Neural Network) and DIR (Deformable Image Registration) in terms of Dice coefficient value and Added Path Length (APL) in voxels. The p-values from a Wilcoxon two-sided signed rank test are given to depict significance of the difference between the approaches.

	ANN DICE	DIR DICE	p-value	ANN APL	DIR APL	p-value
CTV	0.92 ± 0.03	0.95 ± 0.03	<0.001	3109 ± 1642	1835 ± 1621	<0.001
Bladder	0.93 ± 0.07	0.93 ± 0.08	0.60	7250 ± 4234	7236 ± 4287	0.77
Rectum	0.84 ± 0.10	0.88 ± 0.06	<0.001	5041 ± 2666	4170 ± 2920	<0.001
All	–	–	–	15400 ± 6217	13242 ± 6083	<0.001

**Fig. 1:**
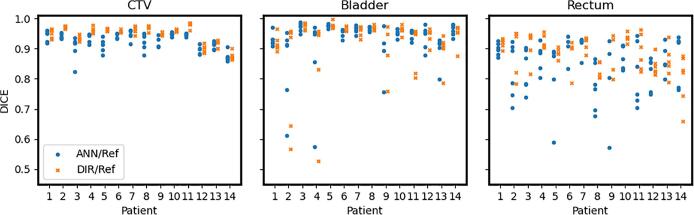
Results from ANN (Artificial Neural Network) (blue circle) and DIR (Deformable Image Registration) (orange cross) compared to reference structures for each patient and all fraction images except the first which was the training image. Results are separated for each patient along the x-axis and presented as Dice coefficient on the y-axis. Each marker represents the result from one fraction image. (For interpretation of the references to colour in this figure legend, the reader is referred to the web version of this article.)

**Fig. 2:**
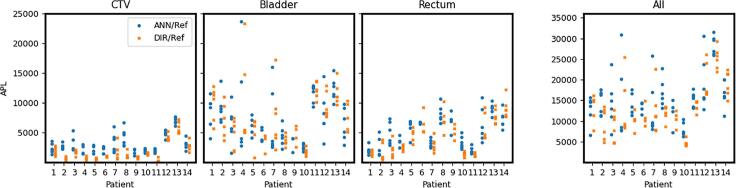
The results from ANN (Artificial Neural Network) (blue circle) and DIR (Deformable Image Registration) (orange cross) compared to reference for each patient and all fraction images except the first which was the training image. Results are separated for each patient along the x-axis and presented as Added Path Length (APL) in voxels on the y-axis. Each marker represents the result from one fraction image. In the rightmost plot each structure contribution is summed. (For interpretation of the references to colour in this figure legend, the reader is referred to the web version of this article.)

However, both metrics varied quite substantially both between and within patients, as depicted in Fig. 1 and Fig. 2 and apparent especially for the rectum.

**Fig. 3:**
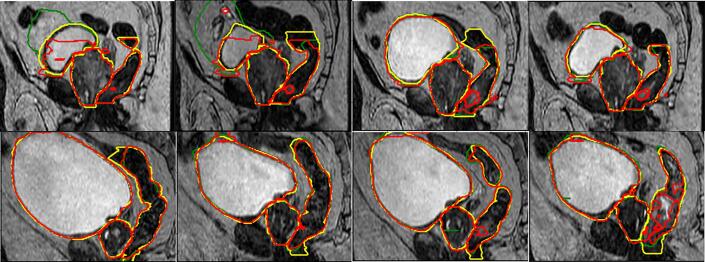
Longitudinal results of patient 2 (top) and 5 (bottom). Yellow structures indicate reference contours, red artificial neural network results and green deformable image registration results. (For interpretation of the references to colour in this figure legend, the reader is referred to the web version of this article.)

For the rectum, variable contrast between the training and test images was generally difficult for the ANN to handle, as depicted in the example results in Fig. 3 where bright areas of the rectum where misinterpreted as belonging to the background.

**Fig. 4:**
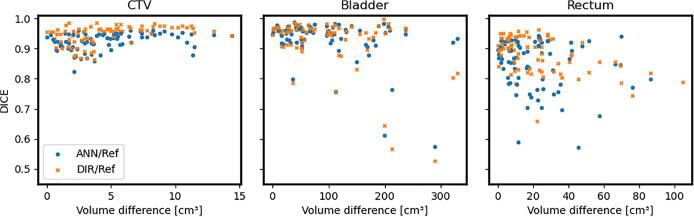
Results in terms of Dice coefficient as a function of volumetric difference for the ANN (Artificial Neural Network) (blue circle) and DIR (Deformable Image Registration) (orange cross). The volumetric difference is taken as the absolute value of the difference in volume between the ground-truth structures on the training images (i.e. first fraction image) and the testing images. Each marker represents the result from one fraction image. (For interpretation of the references to colour in this figure legend, the reader is referred to the web version of this article.)

No tendency of performance degradation due to volumetric differences between training and test data was seen for the CTV, which however had small variation of volume (<15 cm^3^), while the variation in performance was larger for the bladder and the rectum having larger differences in volume, (<350 cm^3^ and <110 cm^3^ respectively), see Fig. 4.

## 4 Discussion

In this work we trained patient specific segmentation ANNs on a single dataset for longitudinal propagation of contours in prostate ultrahypofractionated radiotherapy with an MR-Linac. Despite the minimal amount of training data, the proposed ANN approach produced similar accuracy in terms of both Dice coefficient and APL compared to the DIR approach.

However, as previously mentioned, the ANNs will only work with precision when the inference data is similar to the training data. This inherently causes issues. The most prominent failure of ANN is depicted in Fig. 3, when there was a large variation in contrast between the training image and inference image in the rectum, producing holes in the structure. In case of large volume differences between the structures on the training image and the inference image one could potentially have expected the performance of especially the DIR approach to degrade. As for the CTV, no difference in performance due to volume change seemed to be present for either ANN or DIR, although this could likely be due to the rather small absolute volume changes. Larger volume changes was observed for the bladder and rectum and also a larger spread in performance for both the ANN and DIR approach, however it seems like the volume change was not the only factor contributing to the variation in performance. Noticeable is that in some cases, e.g CTV for patients 12–14, the results between and ANN and DIR were very similar but not fully accurate when compared to the manual ground-truth structures. Similarly, this can be seen in the example image in Fig. 3 for the rectum, where especially the cranial extent of the structure was similar between the ANN and DIR but sometimes not with the ground-truth. Such deviations could potentially be explained, not by the inaccuracy of the approaches, but rather by a variation in definition of the ground-truth structures. It is hence evident that, regardless of approach chosen, the reference data need to be carefully constructed since both approaches, however in very different ways, propagate this information. Due to the usage of 2D-models in the transversal plane, an additional potential explanation for the ANNs inability to distinguish the rectum extent is the processing on a slice-wise basis not considering adjacent slices, noticed in e.g. [22], [23]. Also, obtaining an accurate initial dataset where only minor or no adjustments are required may decrease both the intra- and interobserver variations, (e.g.[24]), hence obtaining more coherent segmentations used for treatment follow-up purposes (e.g. through dose accumulation).

The reference data from the initial plan was here not utilized as the training data for the ANN framework. The reason was that this data was acquired on a different scanner and with different sequence parameters, yielding images with different contrast, resolution and noise than the daily treatment images. Initial training on these images unfortunately did not produce satisfactory results at inference on the daily images, which is an apparent shortcoming of this ANN framework. Potentially some more sophisticated augmentation could have mitigated this issue, such as a histogram matching technique [25]. Also due to the rather stochastic nature of the interfractional variation, a leave-one-out approach could have been utilized, training on images from any of the fractions and applied on the remaining, without losing any generalization, hence strengthening the conclusions. Other longitudinal studies have exploited information in following imaging sessions, in which new images were continuously included [15], [26]. This approach has not been tried in this context but is an evident candidate for evolution of this framework, possibly yielding continuously more accurate models for each fraction. However, this would require manual recontouring of each structure at every fraction if not determined acceptable by the online DIR approach.

The combination of several outputs of a single model has the advantage of simplicity at both training and inference. However, it may potentially be suboptimal, especially when the volume of the segmented structures varies substantially. As in this case, the CTV was substantially smaller than the bladder and rectum and hence the class imbalance may still be of concern for the CTV. A much smaller cropped volume, encompassing solely the CTV with some margin and employing a network onto this volume would potentially have increased the segmentation accuracy. Splitting into different models may also have improved the results.

For future work, a transfer learning approach with a baseline model for the OARs could potentially have improved the results, at least for the rectum segmentation due to the high variation in not only shape but also in contrast. As for the threshold of one voxel for the APL, it was chosen to avoid penalizing structures within clinical acceptance by not requiring a perfect overlap. However, one could argue for different thresholds e.g. for OARs, especially at a distance sufficiently far away from the target and hence to a large degree outside the intended treatment volume. Also, no consideration was given to a potentially more care given to the contouring of the target, which would have implied a higher weight to this structure, or to slice-wise interpolation reducing the number of slices to correct. Here we have focused on evaluating a recontouring scenario, thus emphasizing a measure reflecting this. However, for future work it would be of interest to focus on the dosimetric aspects of different contouring approaches and evaluate their effect of the replanning stage and the dose delivered.

In conclusion, we have trained patient specific ANNs on only a single image set for contouring CTV, bladder and rectum and employed in the context of an adaptive prostate radiotherapy workflow using an MR-Linac. Although a very simple approach, it showed similar performance in comparison to a DIR algorithm optimized for the specific task.

## Declaration of Competing Interest

The authors declare that they have no known competing financial interests or personal relationships that could have appeared to influence the work reported in this paper.
