# Supplementary material for: Patient specific deep learning based segmentation for magnetic resonance guided prostate radiotherapy
Source: Phys Imaging Radiat Oncol. 2022 Jun 3;23:38–42. doi: 10.1016/j.phro.2022.06.001 (PMC9234226; doi:10.1016/j.phro.2022.06.001)

# Supplementary material

## MR-Linac daily workflow for prostate treatment at Uppsala Akademiska Hospital


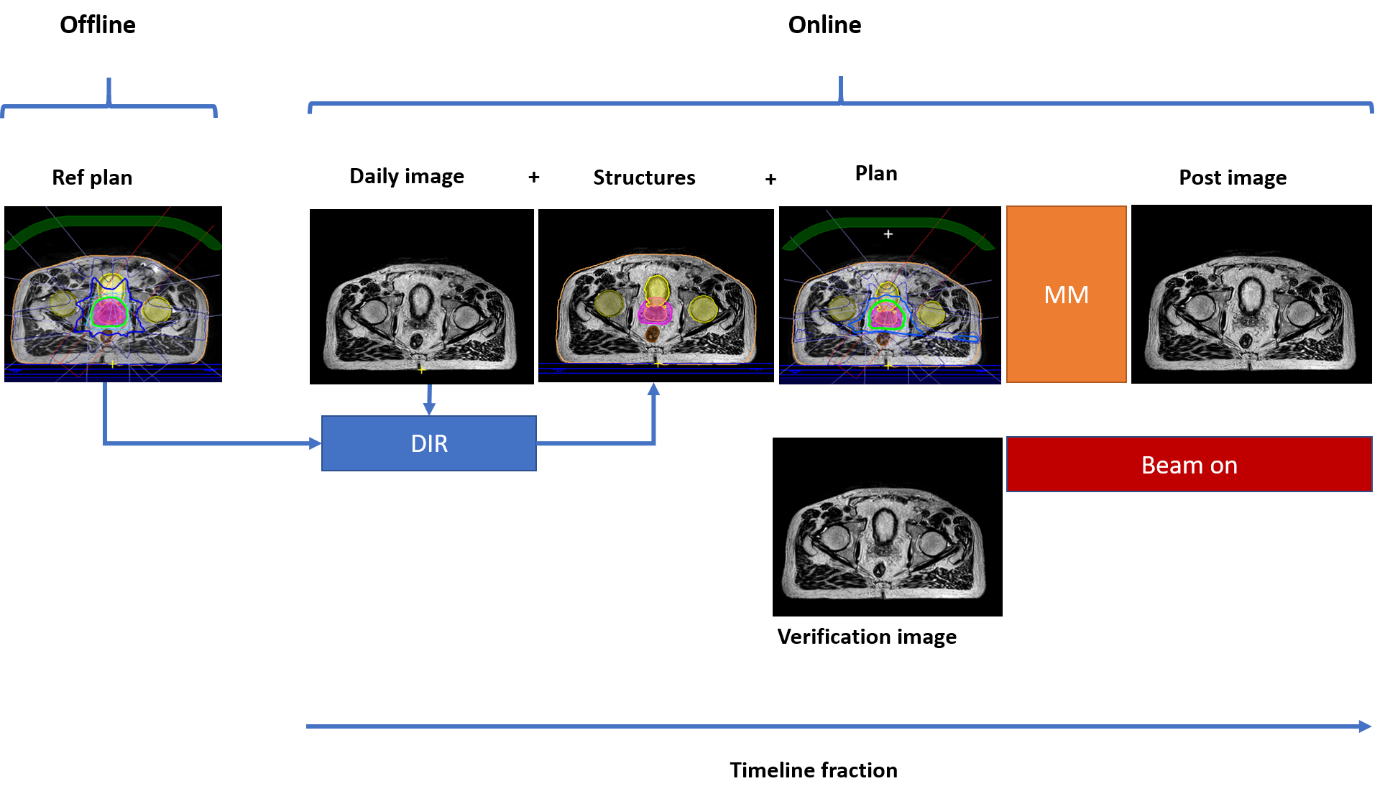


Figure S1. Schematic overview of our MR-Linac workflow with propagated contours through a deformable image registration (DIR) algorithm. During beam on motion monitoring (MM) through cine 2D images are acquired, followed by a full 3D image.

## Model architecture

The model in this work has a u-net shaped architecture (graphical view last in this document). The initial number of feature maps in first (top) level is 8, doubling the number of feature maps in each subsequent lower level after maxpooling, reducing the spatial extent of the feature maps with half in each direction. Alpha dropout with a factor of 0.4 is used in the bottleneck layer. Upsampling in the decoder is performed with transposed convolution. Softmax is applied at the last layer to produce four outputs. Concatenation is performed between the corresponding levels of the encoder and decoder. SeLu (Scaled exponential Linear unit) activation is utilized in all other layers.

The network is implemented and trained in Tensorflow 2.4 with an Nvidia RTX3070-card.

## Performance metrics

Performance metrics for the artificial neural networks (ANNs) on the training data

|  | **ANN**  **Dice** | **ANN Sensitivity** | **ANN Specificity** | **ANN Accuracy** | |  |
| --- | --- | --- | --- | --- | --- | --- |
| CTV | 0.98±0.005 | 0.974±0.001 | 0.999±0 | | 0.999±0 | |
| Bladder | 0.99±0.004 | 0.988±0.006 | 0.999±0 | | 0.999±0 | |
| Rectum | 0.97±0.006 | 0.969±0.013 | 0.999±0 | | 0.999±0 | |

Performance metrics on test data for the artificial neural networks (ANN) and deformable image registration (DIR)

|  | **ANN Sensitivity** | **DIR Sensitivity** | **ANN Specificity** | **DIR Specificity** | **ANN Accuracy** | **DIR Accuracy** |
| --- | --- | --- | --- | --- | --- | --- |
| CTV | 0.931±0.033 | 0.951±0.026 | 0.999±0 | 0.999±0 | 0.999±0 | 0.999±0 |
| Bladder | 0.913±0.099 | 0.932±0.088 | 0.999±0.001 | 0.998±0.004 | 0.996±0.005 | 0.995±0.006 |
| Rectum | 0.815±0.136 | 0.895±0.073 | 0.999±0.002 | 0.998±0.003 | 0.997±0.002 | 0.997±0.003 |


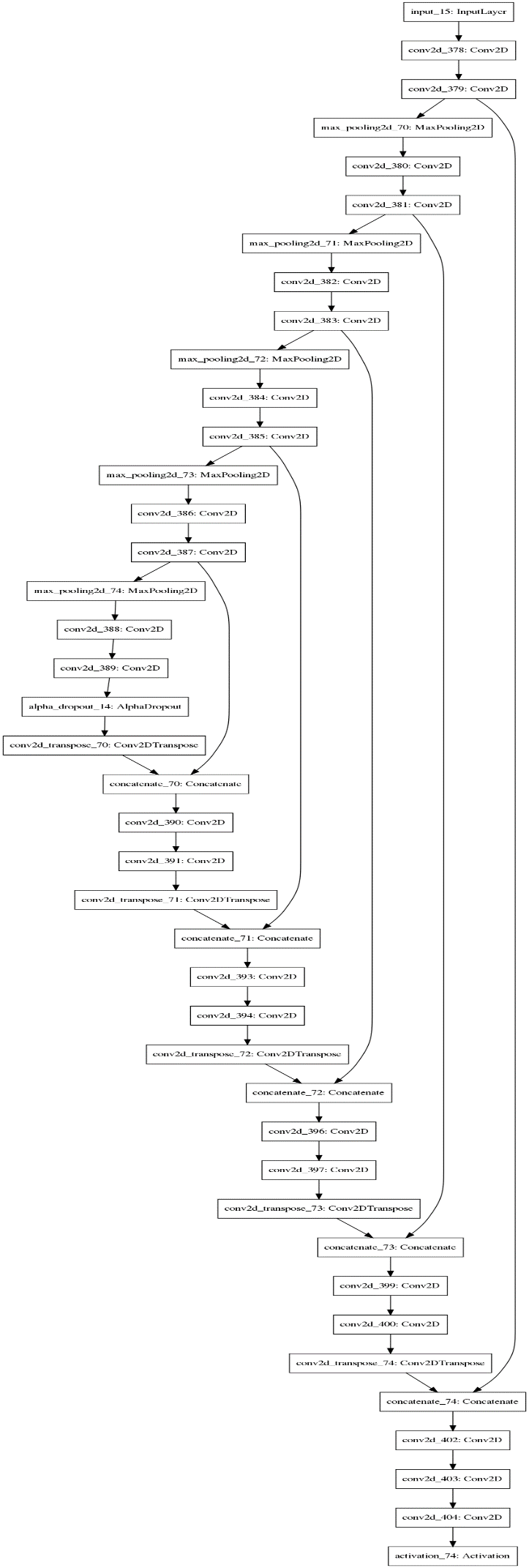

Supplement: Supplementary data 1 [file mmc1.docx]
